# Evaluation of the Therapeutic Effects of Protocatechuic Aldehyde in Diabetic Nephropathy

**DOI:** 10.3390/toxins13080560

**Published:** 2021-08-10

**Authors:** Yu-Teng Chang, Mu-Chi Chung, Chang-Chi Hsieh, Jeng-Jer Shieh, Ming-Ju Wu

**Affiliations:** 1 Institute of Biomedical Sciences, National Chung Hsing University, Taichung 402, Taiwan; t0953337796@yahoo.com.tw; 2 Division of Nephrology, Department of Internal Medicine, Taichung Veterans General Hospital, Taichung 407, Taiwan; mcchung0322@gmail.com; 3 Rong Hsing Research Center for Translational Medicine, National Chung Hsing University, Taichung 402, Taiwan; 4 Ph.D. Program in Translational Medicine, National Chung Hsing University, Taichung 402, Taiwan; 5 Department of Medical Laboratory Science and Biotechnology, Asia University, Taichung 413, Taiwan; 6 Department of Animal Science and Biotechnology, Tunghai University, Taichung 407, Taiwan; cchsieh@thu.edu.tw; 7 Department of Medical Research, Taichung Veterans General Hospital, Taichung 407, Taiwan; 8 College of Medicine, National Chung Hsing University, Taichung 402, Taiwan; 9 School of Medicine, Chung Shan Medical University, Taichung 402, Taiwan; 10 Graduate Institute of Clinical Medical Sciences, School of Medicine, China Medical University, Taichung 404, Taiwan

**Keywords:** diabetic nephropathy, epithelial–mesenchymal transition, protocatechuic aldehyde

## Abstract

Diabetic nephropathy (DN) is one of the most severe chronic kidney diseases in diabetes and is the main cause of end-stage renal disease (ESRD). Protocatechuic aldehyde (PCA) is a natural product with a variety of effects on pulmonary fibrosis. In this study, we examined the effects of PCA in C57BL/KS db/db male mice. Kidney morphology, renal function indicators, and Western blot, immunohistochemistry, and hematoxylin and eosin (H&E) staining data were analyzed. The results revealed that treatment with PCA could reduce diabetic-induced renal dysfunction, as indicated by the urine albumin-to-creatinine ratio (db/m: 120.1 ± 46.1μg/mg, db/db: 453.8 ± 78.7 µg/mg, db/db + 30 mg/kg PCA: 196.6 ± 52.9 µg/mg, db/db + 60 mg/kg PCA: 163.3 ± 24.6 μg/mg, *p* < 0.001). However, PCA did not decrease body weight, fasting plasma glucose, or food and water intake in db/db mice. H&E staining data revealed that PCA reduced glomerular size in db/db mice (db/m: 3506.3 ± 789.3 μm^2^, db/db: 6538.5 ± 1818.6 μm^2^, db/db + 30 mg/kg PCA: 4916.9 ± 1149.6 μm^2^, db/db + 60 mg/kg PCA: 4160.4 ± 1186.5 μm^2^ *p* < 0.001). Western blot and immunohistochemistry staining indicated that PCA restored the normal levels of diabetes-induced fibrosis markers, such as transforming growth factor-beta (TGF-β) and type IV collagen. Similar results were observed for epithelial–mesenchymal transition-related markers, including fibronectin, E-cadherin, and α-smooth muscle actin (α-SMA). PCA also decreased oxidative stress and inflammation in the kidney of db/db mice. This research provides a foundation for using PCA as an alternative therapy for DN in the future.

## 1. Introduction

Chronic kidney disease (CKD) occurs when the structure and function of the kidneys become impaired and are unable to return to normal over several months or years. The occurrence of CKD is mainly due to chronic kidney damage, and loss of function as normal tissue is replaced by interstitial fibrotic tissue [1]. Fibrotic tissue damages normal tissue, preventing its regeneration and function. Common complications of CKD include heart disease and high blood pressure [2]. Clinical evidence suggests that patients can also experience sensory–cognitive impairment and bone disease [3,4]. Another study revealed that CKD can lead to anemia, which then affects the central nervous system, and hyperphosphatemia, which can lead to diseases such as dystrophic calcification and hyperthyroidism [5,6,7].

Diabetic nephropathy (DN) is characterized by urinary albumin excretion, diabetic glomerular lesions, and a reduced glomerular filtration rate in diabetics [8]. In the kidneys of patients with CKD, the abnormal accumulation of collagen, fibronectin, and other proteins causes renal fibrosis. Renal fibrosis leads to ESRD, which requires costly dialysis or kidney transplantation [9]. Studies in patients with type 2 diabetes have demonstrated that during the transition from mild to moderate glomerular sclerosis, macrophages in the glomerulus increased briefly and then decreased again in the advanced stages of kidney disease [10]. In 2006, two patient case reports showed that macrophages in diabetic kidneys were associated with serum creatinine levels, mesenchymal fibroblast accumulation, and interstitial fibrosis scores upon biopsy [11,12]. These findings helped reclassify DN as a chronic inflammatory disease. Urinary monocyte chemoattractant protein-1 and TGF-β are markers for inflammation and fibrosis, respectively [13], and are the most predictive clinical risk factors for progression in DN [14]. Other studies have shown that these markers could be used as targets to predict decreased renal function and progression to proteinuria [15,16,17].

TGF-β is a cytokine with multiple functions, which is omnipresent and necessary for cell survival [18]. Before the chronic stages of nephropathy, the local activation of TGF-β in the affected tissues may play a key role in the process of fibrosis [19]. A previous study found that aldose reductase (AR) affects renal mesangial cells and the renal cortex in diabetic mice, leading to the abnormal regulation of oxidative stress, fibrosis, and epithelial–mesenchymal transition (EMT) through Keap1–Nrf2, Tgf-β1/2 and Zeb1/2 signaling [20]. Furthermore, studies have shown that AR inhibitor (ARI), zopolrestat, and epalrestat are effective against DN [21,22,23]. Protocatechuic aldehyde (PCA) was found to suppress AR activity in the lens of streptozotocin-induced diabetic rats [24]. Therefore, patients with DN may benefit from PCA. 

PCA is a water-soluble phenolic acid compound used in Chinese herbal medicine and reportedly functions as an ARI [25]. It has comprehensive pharmacological activities, including antiproliferation, antioxidation, antilipogenesis, antifibrosis, inflammation and apoptosis inhibition, and cardiomyocyte and nerve cell protection [26]. Recently, research has confirmed that PCA can effectively alleviate inflammation and fibrosis in obstructive nephropathy [27] and inhibit myocardial fibrosis by targeting collagen I [28]. PCA can also inhibit the expression of TGF-β in fibrosis [24] and effectively reverse the process of EMT to prevent pulmonary fibrosis [29]. The purpose of this study was to investigate the effects of PCA on DN-induced fibrosis and development in db/db mice.

## 2. Results

### 2.1. Effect of PCA Treatment on Body Weight, Food Intake, and Water Intake

**Figure 1:**
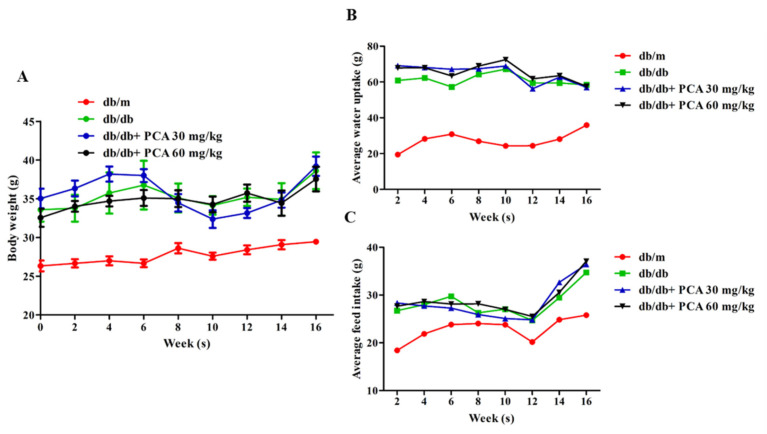
Effects of PCA on mean food intake, water intake, and body weight during the 16-week treatment period. The body weight (**A**), water intake (**B**), and food intake (**C**) of the mice were determined every 2 weeks (*n* = 8). Data are represented as means ± SD. PCA was administered at 30 mg/kg/day and 60 mg/kg/day in db/db mice.

To determine the effect of PCA on DN, db/db mice (approximately 6 weeks old) were orally treated with PCA for 16 weeks. The average body weight of the db/m mice increased from approximately 25 to 30 g during the treatment process, and db/db mice grew from approximately 33 to 39 g (Figure 1A). Water intake among the db/m mice was similar throughout the study and averaged at 20–40 mL/week/animal. The db/db mice maintained a water intake of approximately 60–70 mL/week/animal. Through statistical comparison, the divergence in water intake between db/m and db/db mice was most apparent at 16 weeks (animals aged 20 weeks), coinciding with the onset of severe glycosuria in the latter group. PCA-treated db/db mice did not reduce their water intake (Figure 1B). Food intake in the db/m mice increased during the initial 3 weeks of observation and subsequently stabilized at approximately 25 g/week/animal. The db/db mice consumed 30 g of food with little variation. PCA-treated mice had similar results (Figure 1C). 

### 2.2. PCA Improves Renal Functions in Diabetic Mice

**Figure 2:**
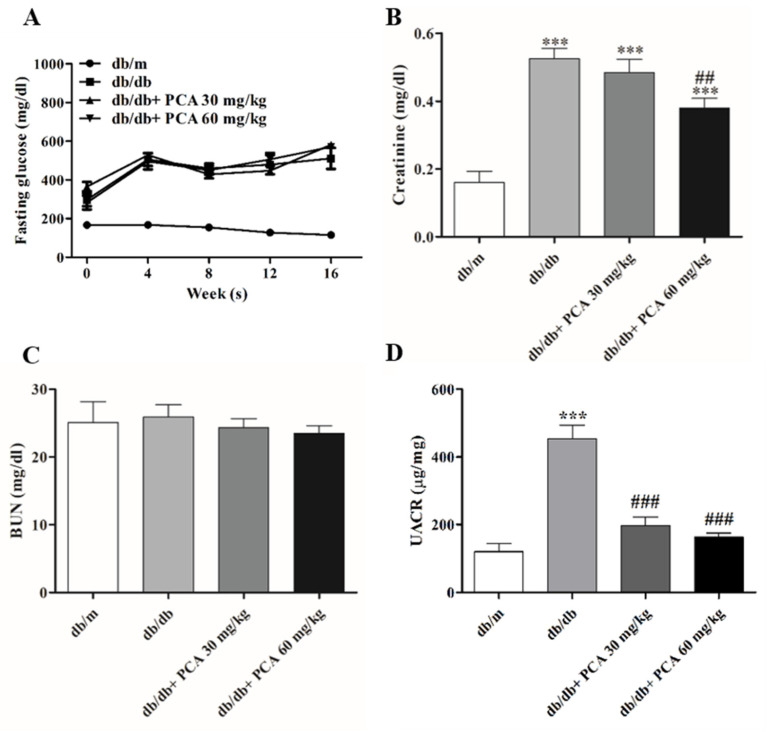
Renal function evaluations of mice. (**A**) Blood glucose levels were detected every 4 weeks (*n* = 8). (**B**,**C**) Quantification of serum creatinine and BUN, respectively (*n* = 8). (**D**) Urine albumin-to-creatinine ratio. The data are presented as means ± SD. *** *p* < 0.001 compared to db/m mice. ## *p* < 0.01, ### *p* < 0.001 compared to db/db mice.

Fasting blood glucose levels were measured at weeks 0, 4, 8, and 16. Compared with the stable blood glucose levels in db/m mice, the glucose levels in db/db mice continuously increased from approximately 300 mg/dL at the start of the experiment (when mice were approximately 6 weeks old) to more than 600 mg/dL (the highest measurement of the glucometer) by the 16th week of the experiment. This suggests that PCA administration had no effect on blood sugar levels (Figure 2A). To assess renal function after PCA treatment, serum creatinine and BUN concentrations were measured to evaluate the effect of PCA in db/db mice. During the study period, the blood creatinine concentration in PCA-treated db/db mice was significantly lower than that in the untreated group (db/m: 0.2 ± 0.1 mg/dL, db/db: 0.6 ± 0.1 mg/dL, db/db + 30 mg/kg PCA: 0.5 ± 0.1 mg/dL, db/db + 60 mg/kg PCA: 0.4 ± 0.1 mg/dL, *p* < 0.001; Figure 2B). The BUN concentration was not significantly different between groups (db/m: 25.1 ± 9.7 mg/dL, db/db: 25.9 ± 5.8 mg/dL, db/db + 30 mg/kg PCA: 24.3 ± 4.0 mg/dL, db/db + 60 mg/kg PCA: 23.5 ± 3.5 mg/dL, *p* = 0.8498; Figure 2C). An elevation in the urinary albumin-to-creatinine ratio (UACR) was noted in db/db mice in the 16th week (Figure 2D); results showed that PCA administration could reduce the UACR (db/m: 120.1 ± 46.1 μg/mg, db/db: 453.8 ± 78.7 μg/mg, db/db + 30 mg/kg PCA: 196.6 ± 52.9 μg/mg, db/db + 60 mg/kg PCA: 163.3 ± 24.6 μg/mg, *p* < 0.001).

### 2.3. PCA Suppresses sCypA and 8-OHdG in Diabetic Mice Urine

**Figure 3:**
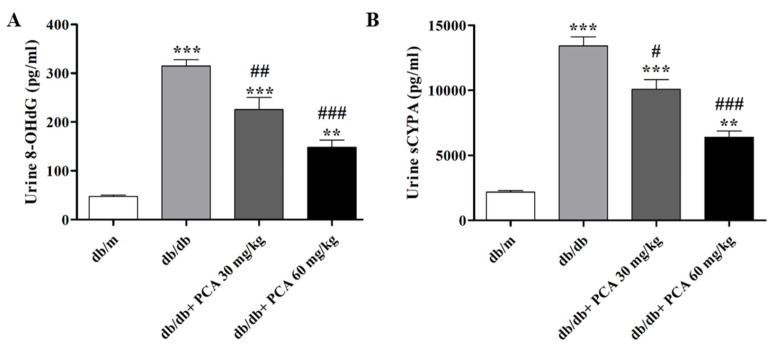
Expressions of 8-OHdG and sCypA from db/db mice urine. (**A**) The expression of 8-OHdG in db/db mice, measured in the urine in the 16th week (*n* = 8). (**B**) The expression of sCypA in db/db mice, measured in the urine in the 16th week (*n* = 8). The data are presented as means ± SD. ** *p* < 0.01, *** *p* < 0.001 compared to db/m mice. # *p* < 0.05, ## *p* < 0.01, ### *p* < 0.001 compared to db/db mice.

Urinary 8-OHdG, which represents DNA damage in early DN, was detected in the 16th week (Figure 3A). Urinary 8-OHdG was significantly increased in db/db mice compared to db/m mice but was suppressed by PCA in a dose-dependent manner (db/m: 46.4 ± 5.6 pg/mL, db/db: 314.8 ± 25.8 pg/mL, db/db + 30 mg/kg PCA: 225.4 ± 49.5 pg/mL, db/db + 60 mg/kg PCA: 148.1 ± 29.3 pg/mL, *p* < 0.001). These results reveal that 8-OHdG decreased with the treatment of 30 and 60 mg/kg/day of PCA. Cyclophilin is a marker for DN [30]. Additionally, the urine sCypA in the db/db mice, which was significantly increased compared to db/m mice, could be decreased with 60 mg/kg/day of PCA (db/m: 2168.4 ± 187.7 pg/mL, db/db: 13,409.3 ± 1201.9 pg/mL, db/db + 30 mg/kg PCA: 10,067.7 ± 1302.7 pg/mL, db/db + 60 mg/kg PCA: 6395.8 ± 814.6 pg/mL, *p* < 0.001; Figure 3B). In summary, we were able to demonstrate that PCA could significantly decrease the expression of 8-OHdG and sCypA compared to db/db mice.

### 2.4. PCA Reduces Kidney Weight and Total Glomerular Area in db/db Mouse Kidneys

**Figure 4:**
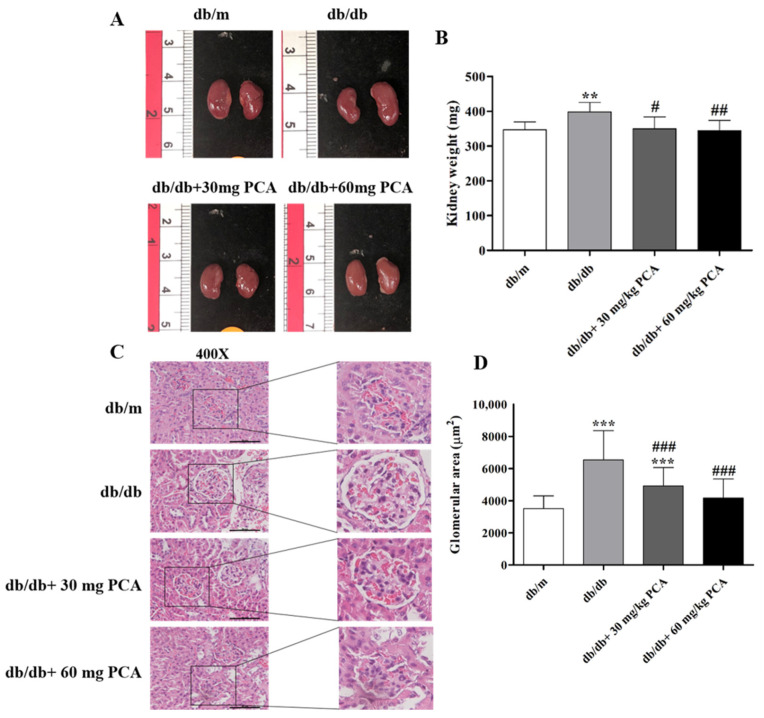
PCA decreases the kidney weight and total glomerular area in db/db mouse kidneys. (**A**) Gross morphologies of kidneys (left panel), (**B**) kidney weight (right panel) (*n* = 8). (**C**) The basic morphology of kidney tissue visualized by H&E staining followed by (**D**) quantitation of the glomerular area (*n* = 5). Scale bar represents 50 μm. The data are presented as means ± SD. ** *p* < 0.01, *** *p* < 0.001 compared to db/m mice. # *p* < 0.05, ## *p* < 0.01, ### *p* < 0.001 compared to db/db mice.

Kidney size was observed after the mice were euthanized, and the mice had no obvious differences in kidney size (Figure 4A, left panel). The kidney weight was measured (Figure 4B, right panel). Compared to db/m mice, db/db mice had a lower weight. However, the db/db mice receiving PCA treatment reduced their kidney weight (db/m: 347.1 ± 22.5 mg, db/db: 398.4 ± 27.4 mg, db/db + 30 mg/kg PCA: 350.0 ± 34.5 mg, db/db + 60 mg/kg PCA: 344.5 ± 29.5 mg, *p* = 0.0029). These results indicate that PCA could recover diabetes-increased kidney weight. To determine the effect of PCA on glomerular hypertrophy in db/db mice, we calculated the glomerular area (Figure 4C,D). The glomerular area of db/db mice was significantly larger than that of db/m mice, but PCA-treated mice had a significantly lower area than untreated db/db mice. Compared to db/m mice, the glomerular area was significantly increased in db/db control mice (db/m: 3506.3 ± 789.3 μm^2^, db/db: 6538.5 ± 1818.6 μm^2^, db/db + 30 mg/kg PCA: 4916.9 ± 1149.6 μm^2^, db/db + 60 mg/kg PCA: 4160.4 ± 1186.5 μm^2^, *p* < 0.001). This suggests that PCA inhibited glomerular hypertrophy.

### 2.5. PCA Inhibits Renal Fibrosis in db/db Mouse Kidneys

**Figure 5:**
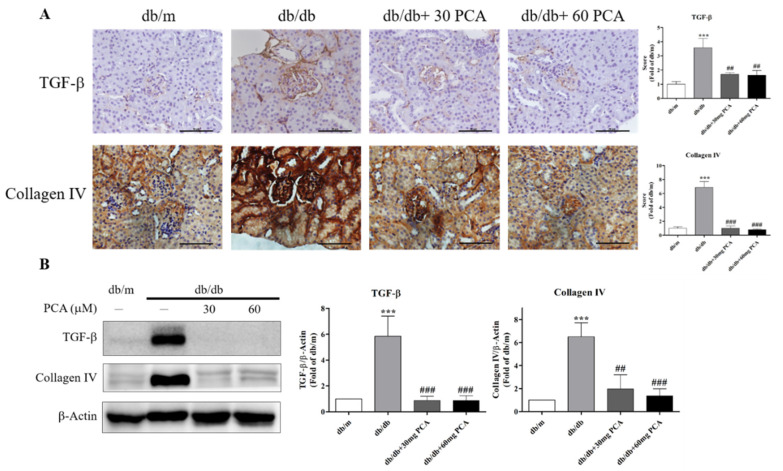
Effects of PCA treatment on renal fibrosis in db/db mouse kidneys. (**A**) Photographs of IHC for fibrosis-related proteins TGF-β and collagen IV (*n* = 5). Scale bar represents 50 μm. (**B**) Western blot analysis of the fibrosis-related proteins TGF-β and collagen IV (*n* = 3). The data are presented as means ± SD. *** *p* < 0.001 compared to db/m mice. ## *p* < 0.01, ### *p* < 0.001 compared to db/db mice.

Increased TGF-β, collagen IV mRNA, and protein levels were observed after the induction of diabetes by STZ in rats [31]. The accumulation of extracellular matrix (ECM) proteins, such as collagen type IV, is common in renal interstitial fibrosis. Substantial research has shown that TGF-β regulates ECM proteins in renal cells [32]. Thus, we examined protein levels using immunohistochemistry (IHC) staining and Western blot. The results demonstrated that PCA inhibited TGF-β and collagen IV expression in db/db mice (Figure 5A,B). These results imply that, compared to untreated db/db mice, PCA-treated db/db mice experienced substantially decreased renal fibrosis.

### 2.6. PCA Inhibits EMT in db/db Mouse Kidneys

**Figure 6:**
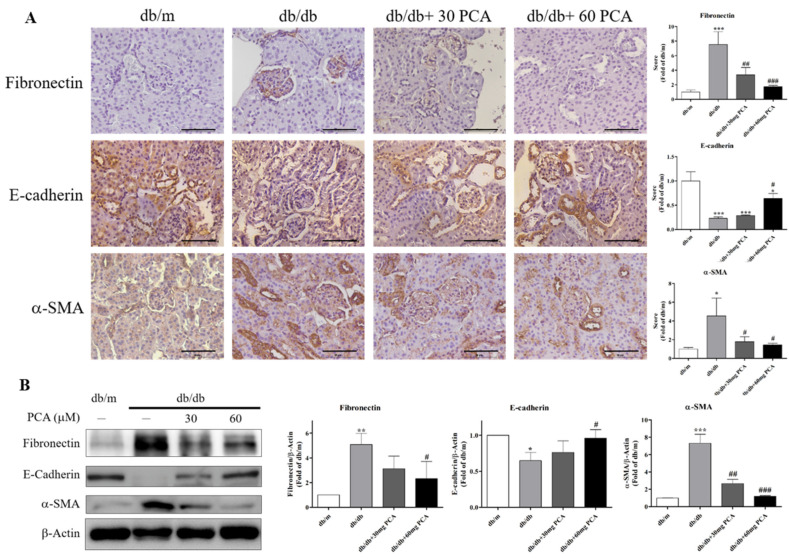
Effects of PCA treatment on EMT in db/db mouse kidneys. (**A**) Photographs of IHC for EMT markers of fibronectin, E-cadherin, and α-SMA (*n* = 5). Scale bar represents 50 μm. (**B**) Western blot analyses were used to detect EMT marker protein levels for fibronectin, E-cadherin, and α-SMA (*n* = 3). The data are presented as means ± SD. * *p* < 0.05, ** *p* < 0.01, *** *p* < 0.001 compared to db/m mice. # *p* < 0.05, ## *p* < 0.01, ### *p* < 0.001 compared to db/db mice.

EMT is the process in which epithelial cells lack adhesion to neighboring cells and transform into migrating invasive cells. Therefore, the protein levels of EMT-related markers were observed using IHC staining and Western blot (Figure 6A,B). The results showed that PCA increased E-cadherin, and decreased fibronectin and α-SMA expression in db/db mice. In conclusion, EMT in the kidneys of db/db mice was markedly recovered by PCA.

### 2.7. PCA Inhibits Expression of AR, Oxidative Stress and Inflammation in db/db Mice Kidneys

**Figure 7:**
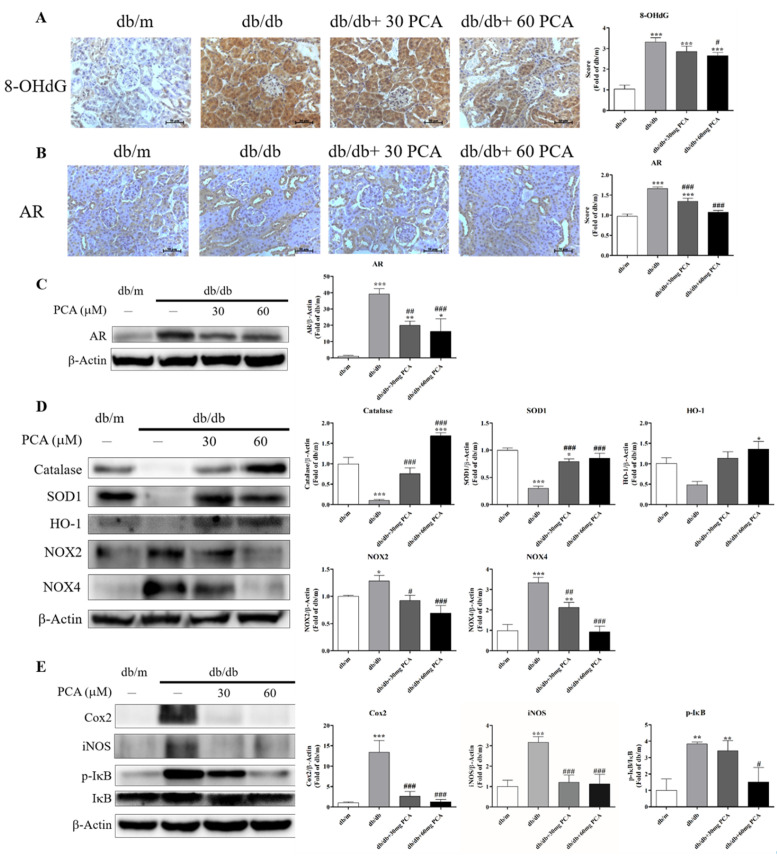
Effect of PCA treatment on expression of AR, 8-OHdG, and markers of oxidative stress and inflammation in db/db mice kidneys. (**A**) Photographs of IHC for 8-OHdG (*n* = 5). Scale bar represents 50 μm. (**B**) Photographs of IHC for AR (*n* = 5). Scale bar represents 50 μm. (**C**) Western blot analyses were used to detect AR protein levels (*n* = 3). (**D**) Western blot analyses were used to detect oxidative stress-related marker protein levels for catalase, SOD1, HO-1, NOX2, and NOX4 (*n* = 3). (**E**) Western blot analyses were used to detect inflammation marker protein levels for Cox2, iNOS, *p*-IκB, and IκB (*n* = 3). The data are presented as means ± SD. * *p* < 0.05, ** *p* < 0.01, *** *p* < 0.001 compared to db/m mice. # *p* < 0.05, ## *p* < 0.01, ### *p* < 0.001 compared to db/db mice.

Urinary 8-OHdG, as a marker of oxidative stress with renal failure, was detected in the kidneys (Figure 7A). Urinary 8-OHdG was significantly increased in db/db mice compared to db/m mice, which could be decreased with PCA. As PCA is an AR inhibitor, we observed the protein levels using Western blot and IHC staining (Figure 7B,C). The results showed that PCA decreased AR expression in the kidneys of db/db mice. As it has been found that PCA can inhibit oxidative stress and, we analyzed oxidative stress markers and inflammation markers using Western blot. The data showed that PCA can increase antioxidant markers (catalase, SOD1, HO-1) and decrease oxidative stress markers (NOX2, NOX4) in the kidneys of db/db mice. Additionally, PCA can suppress inflammation proteins (COX2, iNOS, phospho-IκB) in the kidneys of db/db mice (Figure 7E). In conclusion, PCA can inhibit AR protein expression, 8-OHdG expression, oxidative stress, and inflammation in the kidneys of db/db mice.

## 3. Discussion

Diabetic glomerular disease is characterized by hypertrophy of the kidneys, thickening of the basement membrane, and the gradual accumulation of extracellular interstitium in the glomeruli and tubules. These factors lead to renal fibrosis, glomerular sclerosis, tubular interstitial fibrosis, and, eventually, ESRD [33]. Hence, the development of alternative treatment techniques is essential. As adjuvant therapy, traditional Chinese medicine has demonstrated advantages for patients with different types of fibrosis. Research has discovered that PCA can inhibit the TGF-β1-activated fibrosis [29]. In this study, the relationship between PCA administration and improved renal function was associated with a decrease in creatinine and UACR, although PCA did not affect BUN or blood glucose (Figure 2). Our results also indicated that PCA could suppress 8-OHdG and sCypA after 16 weeks (Figure 3). In summary, PCA effectively reduced diabetes-induced abnormal kidney functions. In addition, we observed that PCA reduced glomerular area in db/db mice treated with PCA for 16 weeks. Our experimental results demonstrated that PCA decreased the kidney weight and glomerular area in db/db mice (Figure 4).

We determined that PCA inhibited TGF-β, and decreased fibrosis and EMT protein levels in the kidneys, which resulted in the restoration of normal levels of diabetes-induced TGF-β and collagen IV expression (Figure 5). Previous research found that hyperglycemia causes morphological changes in podocytes, largely due to EMT. EMT increases the deposition and cross-linking of fibrous ECM proteins, which leads to increased cell–matrix adhesion [34]. In the present study, the results of protein expression analyses revealed the occurrence of EMT in db/db mice, as E-cadherin protein levels decreased, and α-SMA and fibronectin protein levels increased (Figure 6). This indicates that PCA successfully downregulated diabetes-induced renal fibrosis and EMT.

An aldose reductase inhibitor, epalrestat, can decrease AR protein expression in the db/db mice renal cortex. PCA was found to be an AR enzymatic inhibitor (IC50: 0.7 mg/mL), but the effect on protein is uncertain. We proved that PCA can reduce AR protein expression through Western blotting and IHC (Figure 7B,C). As it has been identified that PCA can inhibit oxidative stress and inflammation, we observed their markers using Western blot analysis. (Figure 7D,E). The results revealed that PCA can reduce db/db-induced oxidative stress and inflammation. The oxidative stress-generated reactive oxygen species (ROS) leads to mitochondrial damage that induces endothelial dysfunction [35]. PCA can also protect against oxidative stress and restore endothelial function in diabetic conditions [36]. Additionally, PCA attenuates endothelial dysfunction and atherosclerosis, in vitro and in vivo [37]. Whether or not PCA protects the endothelium of the kidneys in db/db mice requires further investigation.

The transition from hyperglycemia to DN occurs through various pathways, such as the polyol pathway, oxidative stress, and advanced glycation end-product formation [38]. Cellular glucose is mainly metabolized through glycolysis, and increased glucose activates the AR and polyol pathways to produce excess sorbitol [39,40,41]. Since sorbitol in cells does not easily diffuse across cell membranes, glucose and sorbitol accumulate in the cells, along with the downstream metabolite fructose. This promotes an increase in intracellular osmotic pressure, which is considered a pathogenic mechanism that ultimately leads to cell damage [42,43]. The polyol pathway has long been regarded as a key initiating factor of diabetic renal function and structural changes [44,45,46,47], and AR is the rate-limiting enzyme of the polyol pathway [48]. According to previous reports, IHC can weakly detect AR in the renal cortex of nondiabetic patients, but stronger glomerular expression can be observed in samples from DN patients [49,50,51]. Additionally, excess sorbitol can lead to ATP consumption, pro-inflammatory cytokine expression, and oxidative stress. Fructose-fed rats developed moderate tubular interstitial damage and accelerated CKD [52], and fructose supplementation could accelerate kidney disease in residual kidney models [53,54]. 

It should also be noted that this study provides a foundation for studying the role of PCA in diabetic animal experiments. Although our data provide evidence of the protective effect of PCA on DN, the mechanism of this effect still needs to be clarified. Nevertheless, the results from this study support the therapeutic role of PCA in treating DN and should encourage the continued study of this compound for use in clinical applications.

## 4. Conclusions

**Figure 8:**
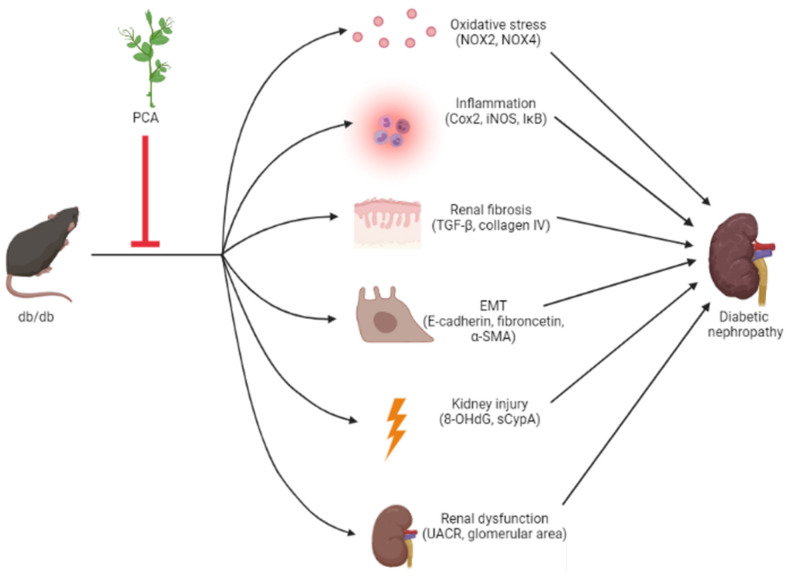
Scheme of effects of protocatechuic aldehyde (PCA) on DN in db/db mice. Collectively, PCA can decrease kidney injury, renal dysfunction, renal fibrosis, EMT, and oxidative stress and inflammation induced by db/db mice. Red inhibition arrows indicate the phenotypes suppressed by PCA.

The results outlined in this study provide evidence for the antifibrotic and anti-EMT effects of PCA in db/db mice, and we suggest PCA as a potential therapeutic agent for moderating DN (Figure 8).

## 5. Materials and Methods

### 5.1. Animals and Treatment (Type 2 Diabetic Animal Model)

Four-week-old male C57BLKS/J db/m and db/db mice were purchased from the National Laboratory Animal Center (Taipei, Taiwan). All mice were individually housed and maintained under environmentally controlled conditions (temperature 22–25 °C, 12 h light/dark cycle, 45–60% humidity). The experimental protocols were approved by a Taichung Veterans General Hospital (TVGH, Taichung, Taiwan) licensing committee (Affidavit of Approval of Animal Use Protocol in TVGH, La-1071589), and the study was conducted according to institutional guidelines. The mice were given a standard sterile rodent chow diet and distilled water ad libitum. At 6 weeks of age, the mice were randomly divided into 4 groups (*n* = 10 in each group initially): (1) db/m mice, (2) db/db mice, (3) db/db + 30 mg/kg PCA, and (4) db/db + 60 mg/kg PCA. The body weight, food intake, and water intake were monitored on a twice-weekly basis. After 16 weeks of PCA administration, the mice were euthanized by isoflurane inhalation at 22 weeks of age. Because of unexpected death in db/db mice, there were 8 mice in each group for further analysis. The blood was collected from the hearts and centrifuged for 10 min at 3000× *g* at 4 °C to obtain the serum, which was stored at −80 °C. The kidney weight was measured after euthanization. The extracted powder of PCA was purchase from Merck Millipore (Carrigtwohill, County Cork, Ireland). It should be noted that 5 out of the 8 mice were randomly chosen for tissue staining, while the other 3 mice were used for Western blotting.

### 5.2. Measurement of Plasma Glucose Levels 

Blood was collected from the mouse tail vein every 4 weeks after overnight fasting. Plasma glucose level was determined with a glucometer and test strips from Accu-Chek (Roche Diagnostics GmbH, Mannheim, Germany), with units in mg/dL.

### 5.3. Renal Function Evaluation

Blood was collected from the left ventricle and centrifuged after euthanization in the 16th week. The plasma was stored at –80 °C for subsequent analyses. In the 16th week, the urea nitrogen (BUN), plasma creatinine, urinary albumin, and urinary creatinine concentrations of the plasma blood were measured by TVGH.

### 5.4. 24 h Urinary Secreted Cyclophilin A and 8-Hydroxy-2′-Deoxyguanosine Detection

Metabolic cages collected urine daily, which was stored in ice immediately, and an ELISA kit (SEA979Mu, USCN Life Science Inc., Wuhan, China) was used to detect secreted cyclophilin A (sCypA). Urinary 8-Hydroxy-2’-deoxyguanosine (8-OHdG), which serves as an established marker of oxidative stress in the kidneys, was detected in 24 h urine using an ELISA kit (CEA660Ge, USCN Life Science Inc.). All results from the ELISA tests were confirmed by performing each test twice.

### 5.5. Protein Extraction and Western Blot Analysis

Total tissue proteins were extracted and lysed using ACK lysis buffer (Thermo Scientific, Waltham, MA, USA) to remove red blood cells, incubated at room temperature for 3–5 min, and centrifuged to collect the pellet. Subsequently, RIPA lysis buffer (Millipore, Billerica) was added, and the samples were stored at −20 °C. 

A quantity of 30 μg of total proteins was resolved by SDS-PAGE and transferred onto polyvinylidene difluoride membranes (Carrigtwohill, County Cork, Ireland). Membranes were incubated with primary antibodies for 18–20 h at 4 °C and then washed with PBST. Secondary antibodies labeled with horseradish peroxidase (HRP) were incubated at room temperature for 1 h and washed with PBS, and then they were detected using an Amersham Imager 680 (Cytiva, Marlborough, MA, USA).

### 5.6. Histological Analysis

The kidney tissues were fixed with 10% formalin, and the lumen was inspected for grossly visible lesions. The right kidney of each mouse was selected for hematoxylin and eosin (H&E) staining using an H&E staining kit (CIS Biotech Inc., Decatur, GA, USA), following the manufacturer’s instructions. In brief, paraffin tissue sections were prepared as usual. Sections were deparaffinized, and slides were hydrated through graded EtOHs with distilled water. Adequate hematoxylin solution was applied to completely cover the tissue section, which was incubated for 1–5 min. The slide was rinsed with distilled water to remove excess stain. Adequate bluing reagent was applied to completely cover the tissue section, which was incubated for 3–5 s, and the slide was rinsed with distilled water. Adequate alcoholic eosin Y solution was added to completely cover the tissue section, which was incubated for 30 s. The slide was rinsed with distilled water to remove excess stain, and the coverslip was air-dried. Additionally, for the quantification of the glomerular area, more than 50 glomeruli were examined to calculate the mean area of the glomerulus for each mouse. The glomerular area was measured using NIS-elements BR software 4.0.

### 5.7. Immunohistochemical Staining 

For immunohistochemistry, the paraffin-embedded kidney sections were deparaffinized in xylene and hydrated in graded alcohol. Subsequently, the paraffin-embedded tissue sections were stained using the Dako REAL EnVision Detection System, Peroxidase/DAB, Rabbit/Mouse (Agilent Technologies, Palo Alto, Santa Clara, CA, USA). Staining was performed after the sections were blocked with normal goat serum and incubated with primary antibodies (or with normal rabbit IgG for the negative control). All staining methods followed the Dako Cytomation EnVision+ Dual Link System-HRP (DAB+) protocol.

### 5.8. Statistical Analysis

Data are presented as means ± SD. One-way ANOVA with Tukey’s test for multiple comparisons was performed using SPSS (IBM SPSS, USA). A *p*-value of <0.05 was considered statistically significant.

## Data Availability

The data presented in this study are available in the article here.
